# Expelling of *Plasmodium falciparum* Sporozoites by *Anopheles stephensi* Mosquitoes During Repeated Feeding

**DOI:** 10.1093/infdis/jiag130

**Published:** 2026-03-03

**Authors:** Chiara Andolina, Geert-Jan van Gemert, Jordache Ramjith, Felix Evers, Wouter Graumans, Rianne Stoter, Karina Teelen, Felix J H Hol, Kjerstin Lanke, Teun Bousema

**Affiliations:** Department of Medical Microbiology, Radboud University Nijmegen Medical Centre, Nijmegen, The Netherlands; Institute of Tropical Medicine, University of Tübingen, Tübingen, Germany; Department of Medical Microbiology, Radboud University Nijmegen Medical Centre, Nijmegen, The Netherlands; Department of Medical Microbiology, Radboud University Nijmegen Medical Centre, Nijmegen, The Netherlands; Department of Medical Microbiology, Radboud University Nijmegen Medical Centre, Nijmegen, The Netherlands; Department of Medical Microbiology, Radboud University Nijmegen Medical Centre, Nijmegen, The Netherlands; Department of Medical Microbiology, Radboud University Nijmegen Medical Centre, Nijmegen, The Netherlands; Department of Medical Microbiology, Radboud University Nijmegen Medical Centre, Nijmegen, The Netherlands; Department of Medical Microbiology, Radboud University Nijmegen Medical Centre, Nijmegen, The Netherlands; Department of Medical Microbiology, Radboud University Nijmegen Medical Centre, Nijmegen, The Netherlands; Department of Medical Microbiology, Radboud University Nijmegen Medical Centre, Nijmegen, The Netherlands; Department of Clinical Research, London School of Hygiene and Tropical Medicine, London, United Kingdom

**Keywords:** interrupted feeding, *Plasmodium falciparum*, sporozoite expelling, artificial skin

## Abstract

It is currently unclear whether repeated mosquito feeding attempts achieve pathogen transmission. We measured *Plasmodium falciparum* sporozoite expelling in *Anopheles stephensi* mosquitoes during 2 interrupted feeding attempts. The number of expelled sporozoites was positively correlated with salivary gland sporozoite load during the first feeding effort (ρ = 0.36, *P* = .0181) but less so during subsequent feeding (ρ = 0.06, *P* = .6811). The median number of expelled sporozoites was of similar magnitude during the first and second feeding event, with a strong correlation between them (ρ = 0.56, *P* = .0002). We conclude that repeated feeding results in repeated sporozoite inoculation at similar intensity.

## Teaser

Malaria-transmitting mosquitoes are regularly interrupted during feeding on human hosts. This study demonstrates that the likelihood that parasites are transmitted and the number of parasites that is injected during feeding are similar for the first and second feeding attempt.

*Plasmodium* parasites are transmitted through the bite of infected female *Anopheles* mosquitoes. Mosquitoes regularly experience interruptions while feeding that may lead to repeated biting, either on the same host or on multiple hosts during the same gonotrophic cycle. In studies where mosquito blood meals were genetically linked to the human blood source, up to 22% of blood meals contained blood from multiple human origins [1, 2], probably acquired during the same night. It is unclear whether repeated biting over a short time frame leads to repeated expelling of pathogens. To allow inoculation, sporozoites must move from the secretory cavity to the salivary duct. Within the salivary duct, sporozoites are organized in parallel or single-file bundles, with only a limited number of sporozoites present at any given time [3]. The inoculum size is highly heterogeneous with a considerable fraction of mosquitoes expelling no sporozoites during a given bite despite infected salivary glands [4, 5]. Local sporozoite supply in the salivary duct may be limited and it is conceivable that repeated bites over a short time frame may result in few or no sporozoites being inoculated. Despite clear implications for malaria transmission dynamics, only 1 study to date examined how interrupted feeding affects sporozoite ejection. In that study, by Ponnudurai and colleagues, *Anopheles stephensi* mosquitoes were fed individually through a mouse skin membrane stretched over human blood. With only 6 mosquitoes used and manual estimation of sporozoite numbers, the study found no evidence for a difference in sporozoite ejection into mouse skin during the first or second feedings [6]. Here, we used a recently developed artificial skin model [4] to further explore sporozoite expelling by individual *Plasmodium falciparum­-*infected mosquitoes that were interrupted during feeding.

## METHODS

### In Vitro Cultures of *P. falciparum* Gametocytes and Mosquito Infections

*Plasmodium falciparum* gametocytes (NF54 strain; West-Africa) were cultured using a standardized protocol. Parasites were maintained at 37°C under a continuously supplied, premixed gas atmosphere consisting of 3% O₂, 4% CO₂, and 93% N₂, and cultured for gametocyte production in a semiautomated tipper system, as previously described [7]. Infection feeding procedures are described elsewhere and involved *An. stephensi* mosquitoes, Nijmegen Sind-Kasur strain [4]. Infection burden was assessed 6 days after feeding by counting the number of oocysts on 1% mercurochrome-stained mosquito midguts. A second uninfected blood meal collected in lithium heparin (Sanquin blood bank; Nijmegen, The Netherlands) was provided 7 days postinfection to synchronize oocyst development [8].

### Mosquito Feeding Experiments

Fifteen days postinfection, individual mosquitoes were collected in small acrylic cages (5 × 5 × 7 cm), covered with netting material on the top and bottom. Mosquitoes were starved for 4 hours prior to feeding experiments. Artificial skin (Integra dermal substitute) was secured to a glass membrane feeder connected to a heated circulating water bath set to 39°C; 100 µL of human blood was pipetted onto the artificial skin's surface (approximately 1.4 cm^2^), and spread evenly for mosquito feeding [4]. The experiments were performed in a secure insectary with the room set at 21°C and 80% relative humidity, to ensure a good heat stimulus for feeding. For the first feeding, the feeder was placed on top of the cage, allowing the mosquito to probe through the membrane for a maximum of 1 minute or until a partial blood meal was observed. Mosquitoes were closely inspected during probing and feeding. Feeding was interrupted once a partial blood meal was observed in the abdomen or if the mosquito was in contact with the membrane for approximately 60 seconds. After the first artificial skin was removed, the same mosquito was allowed to probe on a second artificial skin until it had taken a full blood meal. After the 2 feedings, mosquitoes were dissected under a stereomicroscope, and salivary glands were collected in 1.5-mL Eppendorf tubes containing 180 µL oocyst lysis buffer (NaCl 0.1 M, EDTA 25 mM, TRIS-HCl 10 mM). A scalpel (Dalhausen Präzisa Plus, no 11) was used to cut the artificial skin above the rubber band around the entire feeder. The artificial skin was transferred with DNA-free tweezers to a 1.5-mL Eppendorf tube containing 180 µL lysis buffer (NaCl 0.1 M, EDTA 25 mM, TRIS-HCl. 10 mM), and stored at −70°C. *P. falciparum* sporozoites were quantified by *COX1* quantitative polymerase chain reaction (qPCR) as described elsewhere [4].

### Statistical Analysis

**Figure 1.**
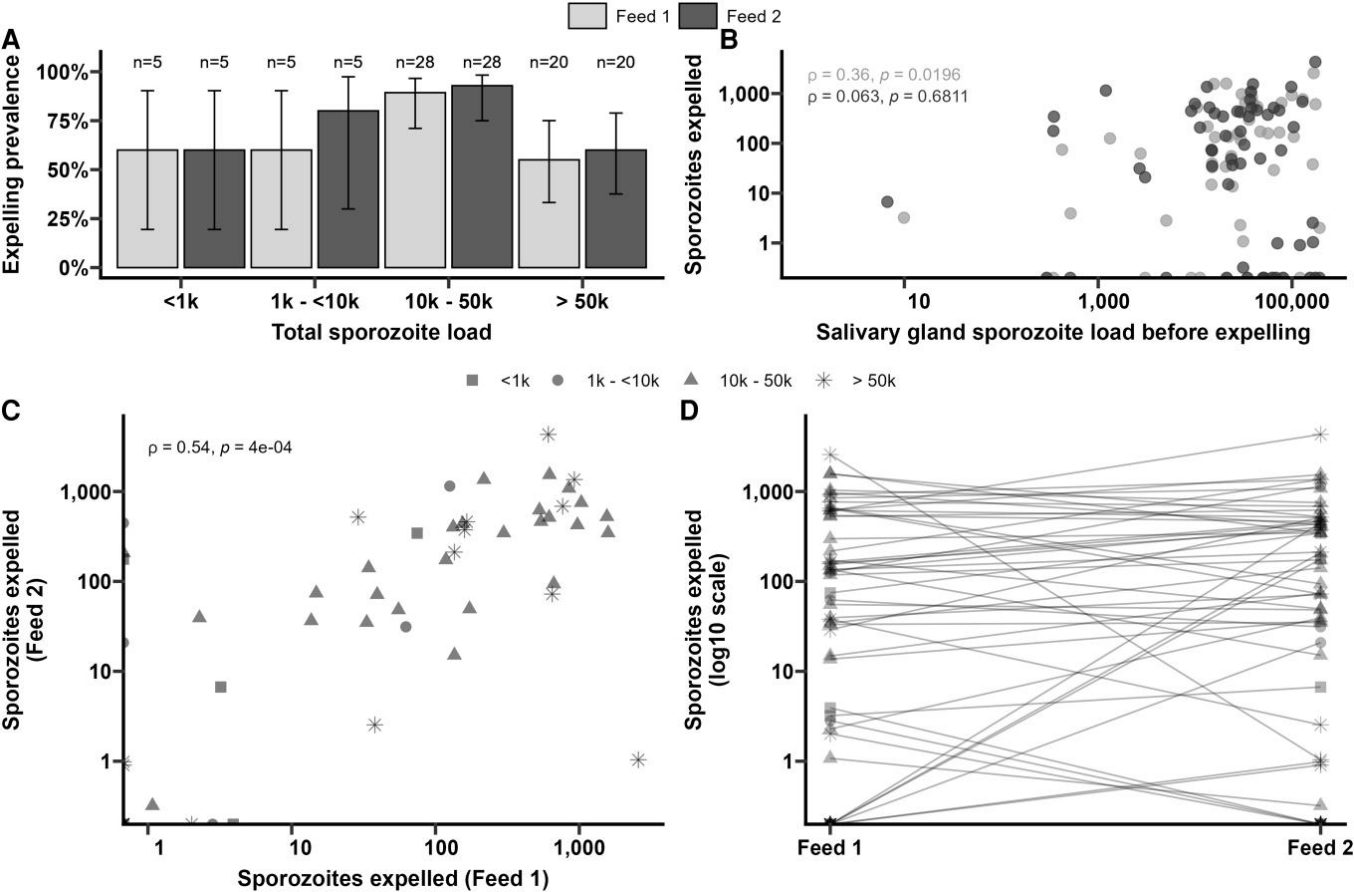
*A*, Total sporozoite loads in artificial skin and salivary glands (x-axis) were binned by infection load (< 1000 [< 1k], 1k–10k, 10k–50k, and > 50k sporozoites) and plotted against the proportion of mosquitoes (%) that expelled sporozoites (y-axis) as estimated from an additive logistic regression model with factors feed 1 and feed 2 categories. *B*, Salivary gland sporozoite load before expelling (sporozoites in salivary glands + sporozoites expelled, x-axis) in relation to the number of expelled sporozoites (y-axis) for feed 1 (ρ = 0.36, *P* = .0181) and feed 2 (ρ = 0.063, *P* = .681). Sporozoite numbers are shown on a log_10_ scale. *C*, The correlation between the number of sporozoites expelled in feed 1 and in feed 2 among mosquitoes that expelled sporozoites in both feeds. Each symbol represents an infected mosquito that fed twice. Different symbols represent mosquitoes from distinct infection load groups. *D*, The total number of sporozoites expelled during both the first and second feeds across all categories. Each pair of points connected with a line represents an individual mosquito. Mosquitoes that never expelled sporozoites are not included in this graph.

Statistical analyses were performed in R (version 4.3.2). Analyses excluded mosquitoes without detectable salivary gland sporozoites. Expelling prevalence was modelled using a logistic regression model with total sporozoite load category and feeding round as fixed factors. Model estimates with 95% CIs were plotted to visualize expelling probabilities (Figure 1*A*). Correlations between (1) salivary gland sporozoite load and expelled sporozoites in feed 1 and feed 2; (2) expelled sporozoites between feed 1 and feed 2; and (3) the corresponding fractions expelled were assessed using Spearman rank correlation (ρ). The fraction expelled was indirectly estimated as the number of expelled sporozoites divided by the sum of expelled sporozoites plus the number of residual salivary gland sporozoites. Paired differences in expelled numbers between feed 1 and feed 2 were evaluated using Wilcoxon signed-rank tests. A 2-sample test for equality of proportions compared the fraction of mosquitoes expelling sporozoites in feed 1 and feed 2. All tests were 2-sided with α = .05.

### Ethics Declarations

Experiments with in vitro cultured parasites and *An*. *stephensi* mosquitoes at Radboud University Medical Center were conducted following approval from the Radboud University Experimental Animal Ethical Committee (RUDEC 2009-019, RUDEC 2009-225).

## RESULTS

Seven independent batches of 20 mosquitoes were used for experiments. Oocyst prevalence on day 6 postinfection was consistently very high, with all dissected mosquitoes harboring oocysts (median oocyst number = 15.5; IQR = 7.25–26.75). Individual mosquitoes were collected in small cages and allowed to probe on artificial skin. Approximately 1 minute after the mosquito started interacting with the artificial skin and a partial blood meal was observed, feeding was interrupted and a second artificial skin was offered to allow the mosquito to feed until repletion. A total of 60 mosquitoes were used for the experiments. Of these, 98% (58/60) had sporozoites detected in either the salivary gland or the skin. One mosquito had no detectable salivary gland sporozoites, but expelled 3 and 7 sporozoites during the first and second feedings, respectively. This mosquito was excluded based on the negative salivary gland and very low skin sporozoite estimates. Finally, 1 mosquito had neither salivary gland sporozoites nor expelled sporozoites, and was thus concluded to be noninfected and therefore excluded from analyses. In the first feed, 72% (42/58) of mosquitoes expelled sporozoites, while in the second feed, 77% (45/58) of mosquitoes expelled sporozoites (*P* = .6680). Among those mosquitoes that did not expel in the first feed (n = 16), 63% (10/16) also did not expel in the second feed. Conversely, among those that expelled in the first feed (n = 42), only 7% (3/42) did not expel in the second feed. Generally, 67% (39/58) of mosquitoes expelled sporozoites in both feeds, 83% (48/58) expelled in at least 1 of the feeds, and 17% (10/58) expelled in neither. Among mosquitoes that expelled sporozoites in both feeds (n = 39), the median number of expelled sporozoites in the first feed was 159 (IQR = 47.3–656.2), while in the second feed it was 346 (IQR = 48.8–521.4) (*P* = .7500). Mosquitoes that failed to expel in both feeds (n = 10) had a median total sporozoite salivary gland load of 56 962 (IQR, 38 503–77 707) (Table 1), which is nonsignificantly (*P* = .2990) higher than the load in mosquitoes that expelled in both feeds (n = 39; median load 30 284; IQR 15 834–51 745). To investigate the relationship between sporozoite expelling and infection burden, mosquitoes were binned into 4 categories based on total sporozoite load (the total number of residual salivary gland sporozoites plus the total number of expelled sporozoites) (Figure 1*A*) [4]. We observed a weak but statistically significant association between salivary gland sporozoite load before expelling and sporozoites expelled in the first feed (Spearman correlation coefficient ρ = 0.36, *P* = .0181; n = 42; Figure 1*B*), indicating that mosquitoes with a higher salivary gland sporozoites load tend to expel more sporozoites during the first feed. In the second feed, no statistically significant association was observed (ρ = 0.06, *P* = .6811; n = 45).

**Table 1.** Sporozoite Load and Inoculum Size in Mosquitoes that Expelled in Repeated Feeds

Expelled Feed 2	Expelled Feed 1	
	Yes	No
Yes	n = 39	n = 6
	SG = 30 284 (15 834–51 745)	SG = 10 165 (4560–55 831)
	E1 = 159 (47.3–656.2)	E1 = 0
	E2 = 346 (48.8–521.4)	E2 = 98.8 (6–200.1)
No	n = 3	n = 10
	SG = 5041 (2780–98 663)	SG = 56 962 (38 503–77 707)
	E1 = 2.8 (2.4–3.4)	E1 = 0
	E2 = 0	E2 = 0

Data are median (IQR).

Abbreviations: E1, number of expelled sporozoites (inoculum size) in the first feed; E2, number of expelled sporozoites in the second feed; n, number of mosquitoes in the category; SG, salivary gland load.

We observed a strong positive association between sporozoites expelled in the first feed and in the second feed (ρ = 0.56, *P* = .0002; Figure 1*C*) that was also evident when paired expelling rates were plotted (Figure 1*D*). This indicates a level of consistency in the number of sporozoites expelled that was also observed when sporozoite expelling was quantified as the fraction of the total sporozoite load (ρ = 0.73, *P* = .0001; Supplementary Figure 3), which was also apparent in individual experiments (Supplementary Figure 4).

## DISCUSSION

We observed a strong correlation between the number of sporozoites expelled during successive feeds and found no evidence that repeated feeding over a short time frame reduces either the likelihood or intensity of sporozoite inoculation. These findings suggest that repeated feeding can result in repeated transmission events of similar magnitude.

Before each gonotrophic cycle a mosquito may take 1 or more blood meals. Low levels of salivary apyrase, an antiplatelet aggregation enzyme, may lead to prolonged probing time and incomplete blood meals, plausibly increasing the biting rate [9]. The consequences of repeated feeding on the likelihood that malaria parasites are transmitted remain largely unstudied. An early study by Rosenberg, in which mosquitoes were attached to a glass slide and forced to salivate into mineral oil, indicated that the majority of *P. falciparum* sporozoites are expelled at the onset of salivation and longer salivation has diminishing returns for transmission potential [10]. In contrast, for *Plasmodium berghei* it was reported that transmission potential may persist throughout multiple probing attempts [11]. We observed that mosquitoes that expelled sporozoites during 1 feeding attempt were highly likely to do so again during a subsequent attempt, with comparable inoculum sizes. This is in line with a limited number of experiments where single infected mosquitoes that were interrupted during feedings through a mouse skin membrane showed no evidence of depletion [6]. Our data further indicate that even mosquitoes with a relatively low infection burden (10 000–50 000 sporozoites in the salivary glands, reflecting approximately 2–10 oocysts [4]), can effectively transmit malaria to multiple hosts within a short time frame.

Intriguingly, repeated biting may be increased in infected mosquitoes. A study in Tanzania investigating whether Anopheles *gambiae* infected with *P. falciparum* sporozoites fed on more hosts than uninfected mosquitoes, found that 22% of infected mosquitoes took multiple blood meals compared to only 10% of uninfected mosquitoes [12]. Together with our finding of a high likelihood of successful inoculation during repeated bites, this suggests that a considerable proportion of infected mosquitoes may transmit *P. falciparum* multiple times during a single night. Interestingly, we observed that mosquitoes with high sporozoite loads (median 56 962) failed to expel sporozoites in both feedings. Similar observations have been made in mosquitoes with a very high number of sporozoites in the salivary glands, where only a small number of sporozoites were expelled [4, 5, 13]. The complex architecture of salivary glands [13] and potential damage to this architecture following high-intensity infection might influence expelling efficiency.

Our study has several limitations. Our modest sample size was insufficient to detect subtle differences but the fact that we observed a numerically higher inoculum size in the second feeding attempt gives us confidence that we can reject our hypothesis that the number of expelled sporozoites declines during repeated feeding. We also did not standardize the duration of feeding; the second feeding may thus have been longer, and it is possible, albeit not proven, that this results in a higher inoculum size [3, 5]. Lastly, although our sporozoite loads were higher than those typically observed in natural infections, high salivary gland sporozoite loads have also been reported in field-collected *Anopheles* mosquitoes [14, 15]. Future studies might examine whether our findings can be extrapolated to mosquitoes with low infection burdens.

We conclude that repeated feeding attempts frequently lead to repeated sporozoite inoculation and provide further evidence that mosquito salivary gland load is a relevant determinant of inoculum size.

## Supplementary Material

jiag130_Supplementary_Data

## Supplementary Data

Supplementary materials are available at *The Journal of Infectious Diseases* online (http://jid.oxfordjournals.org/). Supplementary materials consist of data provided by the author that are published to benefit the reader. The posted materials are not copyedited. The contents of all supplementary data are the sole responsibility of the authors. Questions or messages regarding errors should be addressed to the author.
