# Supplementary material for: Expelling of Plasmodium falciparum Sporozoites by Anopheles stephensi Mosquitoes During Repeated Feeding
Source: J Infect Dis. 2026 Mar 3;233(6):e1465–9. doi: 10.1093/infdis/jiag130 (PMC13271377; doi:10.1093/infdis/jiag130)
Supplement: jiag130_Supplementary_Data [file jiag130_supplementary_data.docx]

**Supplementary Results**

**Supplementary Table 1: Primer and probe sequence used in COX-I sporozoite qPCR.**

| Target | Primer Type | Sequence |
| --- | --- | --- |
|  | Primer-fw (5’-3’) | CATCAGGAATGTTATTGCTAACAC |
|  | Primer- rv (5’-3’) | GGATCTCCTGCAAATGTTGGGTC |
|  | Probe (5’-3’) | 6FAM-ACCGGTTTTAACTGGAGGAGTA-BHQ1 |


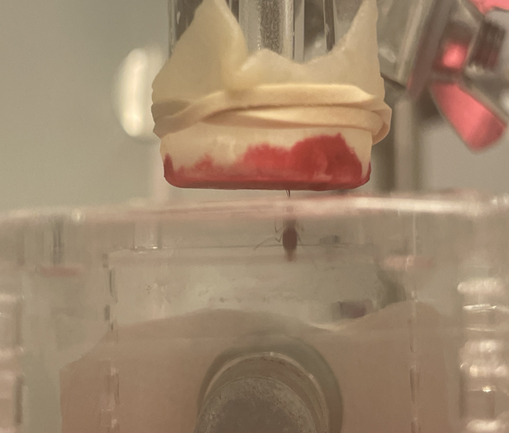
 **
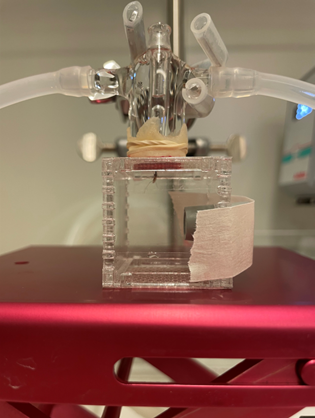
**

**Supplementary Figure 1.** *An. stephensi* mosquito feeding on artificial skin covering a glass feeder connected to a circulating water bath set at 37C.

**Supplementary Figure 2.** Scatter plot showing the distribution of oocyst intensity per mosquito on day 6 post infection across 7 experimental infections. 10 mosquitoes were dissected per experiment Colours indicate infection batches: blue = first, red = second, green = third, purple = fourth, orange = fifth, pink = sixth, and light blue = seventh. The bar represents the mean.

**
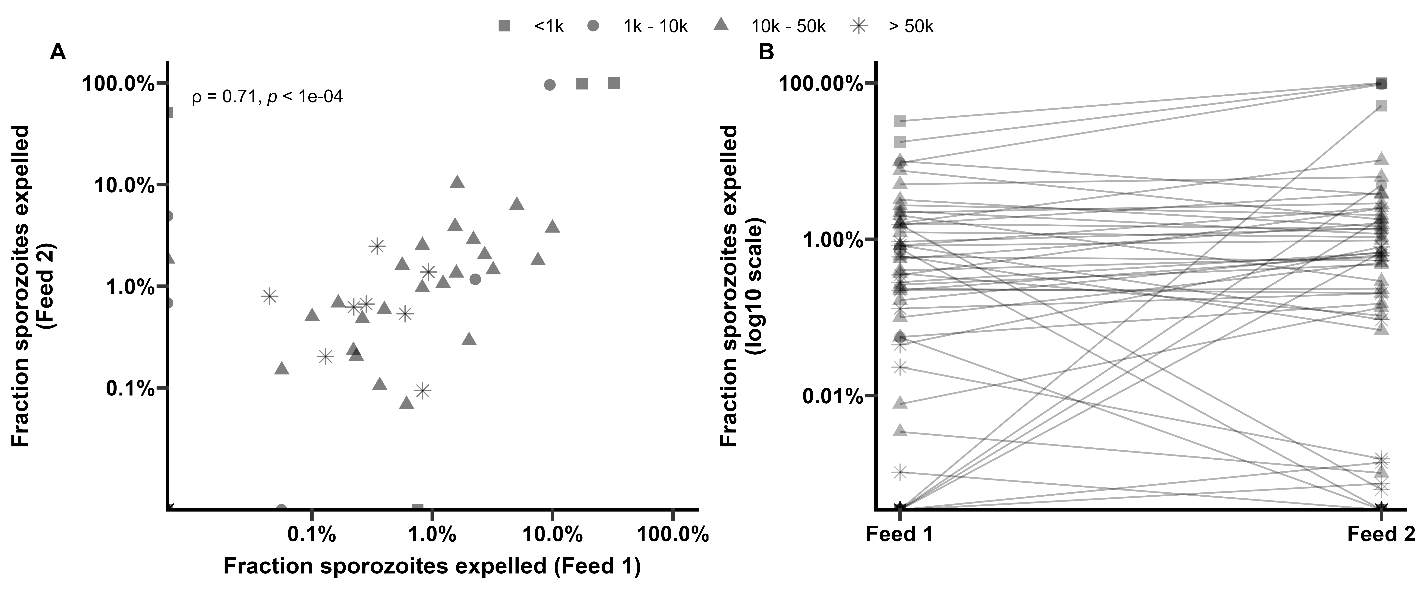
**

**Supplementary Figure 3. Correlation between the fraction of sporozoites expelled during the first and second blood feeds. A.** Each point represents an individual mosquito, with the fraction of sporozoites expelled during Feed 1 (x-axis) plotted against the fraction expelled during Feed 2 (y-axis), both shown on a logarithmic scale. Different symbols (triangles, squares, asterisks, circles) correspond to distinct infection groups. **B.** Each connected line represents one mosquito, showing the fraction of sporozoites expelled during Feed 1 and Feed 2 on a log 10 scale. Symbols denote different infection groups, as in Panel A.

**
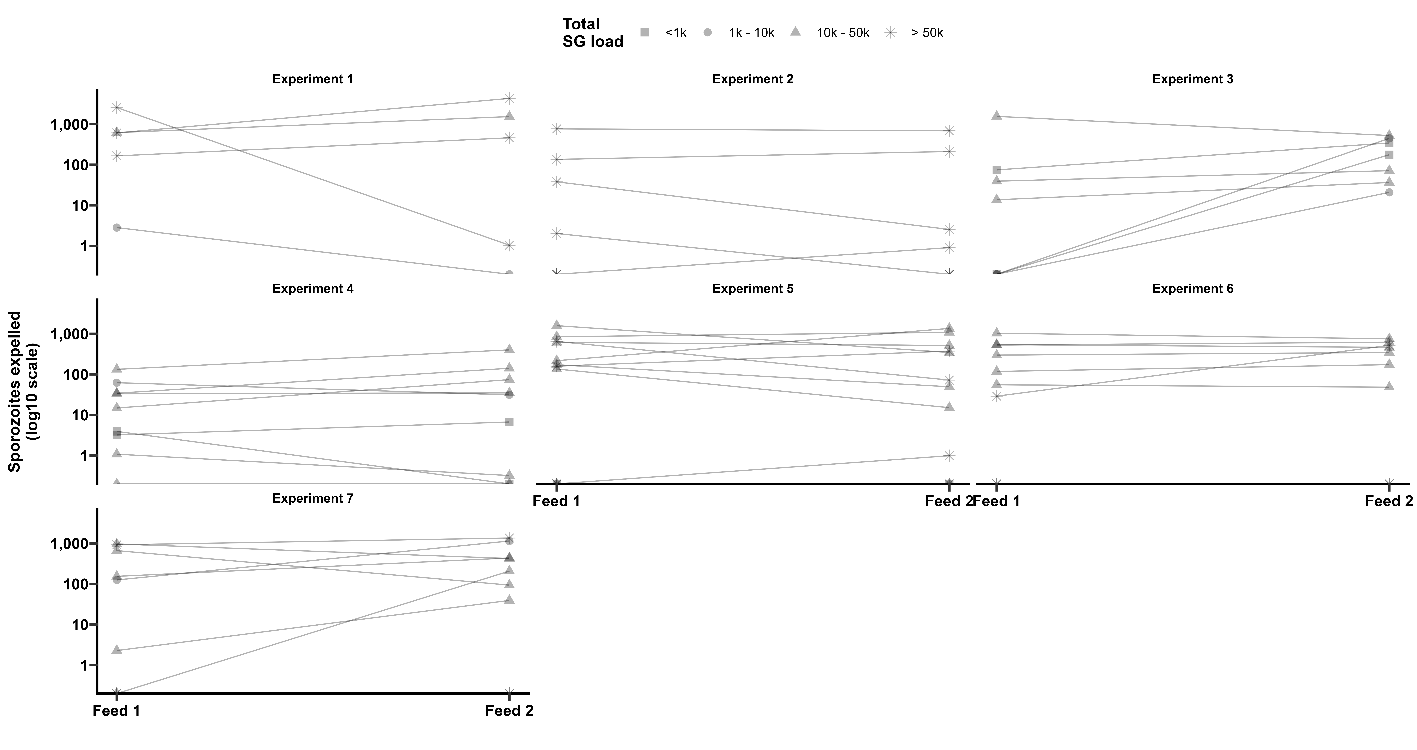
**

**Supplementary Figure 4. The number of sporozoites expelled during the first and second blood feeds.** Each point represents an individual mosquito, with the number of sporozoites expelled during Feed 1 plotted against the number expelled during Feed 2. Different symbols (triangles, squares, asterisks, circles) correspond to distinct infection groups and data are presented for each of the 7 experiments/mosquito batches separately. There is no consistent trend of higher/lower number of expelled sporozoites in Feed 1 versus Feed 2.
